# A Novel LncRNA MASCC1 Regulates the Progression and Metastasis of Head and Neck Squamous Cell Carcinoma by Sponging miR-195

**DOI:** 10.3390/cancers15245792

**Published:** 2023-12-11

**Authors:** Yujia Wang, Zhen Qin, Yiwen Chen, Yunfei Zheng, Lingfei Jia

**Affiliations:** 1 Department of Oral and Maxillofacial Surgery, Peking University School and Hospital of Stomatology, Beijing 100081, China; 2111210411@stu.pku.edu.cn (Y.W.); 2311110613@stu.pku.edu.cn (Z.Q.); 2211210500@stu.pku.edu.cn (Y.C.); 2 Department of Orthodontics, Peking University School and Hospital of Stomatology, Beijing 100081, China; 3 Department of Central Laboratory, Peking University School and Hospital of Stomatology, Beijing 100081, China; 4 National Center of Stomatology & National Clinical Research Center for Oral Diseases & National Engineering Laboratory for Digital and Material Technology of Stomatology, Beijing 100081, China

**Keywords:** head and neck squamous cell carcinoma (HNSCC), lncRNA, metastasis associated squamous cell carcinoma 1 (MASCC1), miRNA, miR-195, progression, metastasis

## Abstract

**Simple Summary:**

LncRNAs are involved in HNSCC carcinogenesis, progression, and metastasis. In this study, we identified a novel lncRNA, named *MASCC1* (Metastasis-Associated Squamous Cell Carcinoma 1), which was highly expressed in metastatic HNSCC and correlated with poor prognosis. In vitro, MASCC1 knockdown (KD) inhibited HNSCC cell proliferation, migration, invasion, and tumor sphere formation while promoting apoptosis. In vivo, MASCC1 KD suppressed HNSCC tumor formation and lymph node metastasis. Mechanistically, MASCC1 acted as a competing endogenous RNA (ceRNA) to sponge miR-195, regulating Cyclin D1, BCL-2, and YAP1 expression. Moreover, miR-195 overexpression rescued the effects of MASCC1 overexpression on the biological behaviors of HNSCC in vitro and in vivo. Our results show that lncRNA MASCC1 functions as an oncogene and is involved in HNSCC progression and metastasis, providing a potential target for HNSCC treatment.

**Abstract:**

The altered expression of long noncoding RNAs (lncRNAs) is associated with human carcinogenesis. We performed a high-throughput analysis of lncRNA expression in strictly selected pairs of metastatic head and neck squamous cell carcinoma (HNSCC) and non-metastatic HNSCC samples. We identified a novel lncRNA, which was highly expressed in metastatic HNSCC, named Metastasis Associated Squamous Cell Carcinoma 1 (MASCC1), for further study. Using qRT-PCR, we further compared MASCC1 expression in 60 HNSCC samples. The results show that high expression of MASCC1 in patients with HNSCC was related to poor prognosis. In vitro, MASCC1 knockdown (KD) inhibited HNSCC proliferation, migration, invasion, and tumor sphere formation, while promoting apoptosis. In vivo, MASCC1 KD inhibited HNSCC growth and lymph node metastasis. Mechanistically, MASCC1 acted as a competing endogenous RNA (ceRNA) by binding to miR-195, subsequently regulating the expression of Cyclin D1, BCL-2, and YAP1. Moreover, miR-195 overexpression rescued the effects of MASCC1 on the biological behaviors of HNSCC. Taken together, our results suggest that *MASCC1* is a novel oncogene that can predict the prognosis of patients with HNSCC and is a potential therapeutic target for HNSCC intervention.

## 1. Introduction

Head and neck squamous cell carcinoma (HNSCC), mostly located in the oral cavity, pharynx, and larynx, is the sixth most common cancer worldwide [1,2], accounting for 90% of head and neck cancers [3]. HNSCC is an aggressive malignant tumor with rapid proliferation, cervical lymph node metastasis, recurrence, and poor prognosis [1,4,5]. Despite advances in multimodal treatments, the 5-year survival rate has not improved [1,3,6]. The lack of efficient diagnostic and prognostic biomarkers is mostly responsible for the high mortality rates [1,7]. Moreover, a lack of information on the molecular carcinogenesis and genetics of HNSCC has hampered the development of novel therapeutic strategies [6]. Thus, early diagnosis and effective therapy are important for HNSCC treatment, and understanding the molecular pathways underlying its carcinogenesis and progression would help the prevention, diagnosis, and treatment of HNSCC.

Differentially expressed genes identified from recent cancer profiling studies have improved tumor diagnosis and therapy. However, the human transcriptome comprises not only protein-coding RNAs, but also many noncoding RNAs (ncRNAs) with structural, regulatory, or unknown functions [8,9]. Long noncoding RNAs (lncRNAs) are more than 200 nucleotides in length and regulate transcription, nuclear structure organization, and protein modulation [8,10]. Several lncRNAs, such as MIR31HG [11], LINC00460 [12], and HOTTIP [13], are dysregulated in HNSCC and are believed to contribute to its development and progression. Other tumor-related lncRNAs might also have value for HNSCC diagnosis and therapy.

Here, we compared the expression of different lncRNAs between metastatic HNSCC and non-metastatic HNSCC samples and identified a novel lncRNA, Metastasis Associated Squamous Cell Carcinoma 1 (MASCC1), whose expression was increased in metastatic HNSCC. We found that MASCC1 is involved in the progression and metastasis of HNSCC by sponging miR-195, providing a potential target for HNSCC therapy.

## 2. Materials and Methods

### 2.1. Clinical Samples

As approved by the Ethics Committee of the Peking University School of Stomatology, 60 patients who were admitted to the Department of Oral and Maxillofacial Surgery at the Peking University School of Stomatology (Beijing, China) between 2008 and 2011 were enrolled in the present study. HNSCC tumor tissues of the patients were collected after informed consent was obtained. The patients had not received any other treatment before the radical surgery. Fresh tumor tissues and non-tumoral tissues that were at a distal distance of at least 1.5 cm away from the tumor margin were collected after the resection. Then, the samples were snap-frozen in liquid nitrogen and stored at −80 °C until use [14]. A pathologist then carefully dissected the samples, and hematoxylin and eosin (H&E) staining was performed to obtain a template that identified areas containing at least 70% tumor or normal cells. Then, the samples that met the criteria were prepared for the following analysis. 

### 2.2. Microarray Analysis

Microarray analysis was performed using four pairs of metastatic HNSCC and matched non-metastatic HNSCC samples. Total RNAs were extracted using TRIzol (Thermo Fisher Scientific, Waltham, MA, USA). RNA was transcribed into fluorescent (Cy5 and Cy3-dCTP) labeled cDNA. Then, hybridization and signal acquisition on the Human LncRNA Array ver. 1.0 (123135K, Roche Nimblegen, Mannheim, Germany) containing 30,618 lncRNA transcripts were performed. The raw data were extracted using NimbleScan software (ver. 2.5; Roche Nimblegen), and the signal intensities were normalized. Differentially expressed lncRNAs were defined based on the fold change (FC) and *p*-value (FC > 3; *p* < 0.05). The raw and processed microarray data were deposited in the Gene Expression Omnibus database in the National Center for Biotechnology Information with the accession number GSE246104.

### 2.3. qRT-PCR

Total RNAs were extracted from tumor tissues using TRIzol, and then 500 ng of RNA was reverse transcribed to cDNA using a reverse transcription kit (Takara, Dalian, China). A SYBR Green kit (Roche Diagnostics, Mannheim, Germany) was used to quantify the cDNA using quantitative real-time PCR, which was conducted at 95 °C for 10 min, followed by 40 cycles of 95 °C for 15 s and 60 °C for 1 min using the ABI Prism 7500 real-time PCR System (Applied Biosystems, Foster City, CA, USA). The primers for qRT-PCR are listed in Table S1. The relative expression level was calculated using the comparative threshold method.

### 2.4. 5′- and 3′-Rapid Amplification of cDNA Ends (RACE)

We used the SMART™ RACE cDNA Amplification Kit (Clontech, Mountain View, CA, USA) to obtain the full-length MASCC1 following the manufacturer’s instructions. Briefly, total RNA (10 µg) was isolated and treated with TAP enzyme. Then, the RNA was submitted to 5′-RACE adapter ligation and reverse transcription. With primers recognizing the 5′ end of MASCC1, nested PCRs were performed. Similarly, a 3′-RACE adapter and primers specific to the 3′ end of MASCC1 were used for 3′-RACE [15]. The sequences of primers are listed in Table S2.

### 2.5. Nuclear and Cytoplasmic Fraction Isolation

The nuclear and cytoplasmic fractions were extracted using a Nuclear and Cytoplasmic Protein Extraction Kit (Beyotime, Beijing, China). HNSCC cells were washed using phosphate-buffered saline (PBS) and Cytoplasmic Extraction Reagent A, B, and Nuclear Extraction Reagent were added in that order, followed by vortexing and centrifugation. The cytoplasmic fraction from the supernatant and the nuclear fraction from the pellet were separated. Then, RNAs were extracted using TRIzol, followed by qRT-PCR using the primers listed in Table S1.

### 2.6. RNA Fluorescence In Situ Hybridization (FISH)

HNSCC cells were fixed in 4% paraformaldehyde, washed using PBS, and then permeabilized with PBS containing 0.5% Triton X-100 for 5 min at 4 °C. After incubating with a mixed blocking solution and pre-hybridization buffer at 37 °C for 30 min, the cells were hybridized with h-U6 FISH Probe Mix (RiboBio, Guangzhou, China), h-18S FISH Probe Mix (RiboBio), and a lncRNA FISH Probe Mix (RiboBio) using a FISH Kit (RiboBio) on glass chamber slides at 37 °C overnight. The cells were washed using 4×, 2×, and 1× saline sodium citrate (SSC) buffer (Beyotime) at 42 °C for 5 min followed by 4′,6-diamidino-2-phenylindole (DAPI) staining for 10 min. All images were captured under a fluorescence microscope.

### 2.7. Cell Culture and MASCC1 Knockdown

CAL27 and SCC15 (ATCC, Manassas, VA, USA), the human HNSCC cell lines, were cultured in Dulbecco’s modified Eagle’s medium (DMEM) supplemented with 10% fetal bovine serum (FBS) at 37 °C in a humidified atmosphere containing 5% CO_2_. The culture medium contained 1% antibiotics (streptomycin and penicillin). After reaching 60% confluence, the cells were transfected with siNC (Negative Control), siMASCC1-1 or siMASCC1-2 (Integrated Biotech Solutions Co., Shanghai, China) using Lipo8000^TM^ Transfection Reagent (Beyotime). Lentivirus-based short hairpin RNA (shRNA) was used to knock down MASCC1. shNC (Integrated Biotech Solutions Co.) or shMASCC1 (Integrated Biotech Solutions Co.) was transfected into HNSCC cells.

### 2.8. MASCC1 and miR-195 Overexpression

The ~383-bp MASCC1 sequence was synthesized and inserted into vector pcDNA3.1-lncRNA (Integrated Biotech Solutions Co.) downstream of the CMV promoter. Recombinant lentiviruses based on pLL3.7, including pLL3.7-NC and pLL3.7-MASCC1, contained the green fluorescent protein (GFP) gene. After HNSCC cells were infected with either pLL3.7-NC or pLL3.7-MASCC1, cells were screened and sorted using fluorescence-activated cell sorting (FACS) based on the expression of GFP, which indicated the presence of the plasmids. Then, the sorted cells were used as the stable negative control (NC) or MASCC1 overexpression cells. The screening of miR-195 overexpression lentivirus (Integrated Biotech Solutions Co.)-infected cells followed the same process as that described for MASCC1.

### 2.9. Cell Counting Kit 8 (CCK-8) Assay

The proliferation of HNSCC cells was evaluated using a CCK-8 assay (Dojindo, Shanghai, China). After transfection, the cells were added to 96-well plates (2 × 10^3^ cells/well). Then, 10 μL of CCK-8 reagent was added to each well at 0, 24, 48, 72, and 96 h, and incubated at 37 °C for 1 h before detection. The absorbance at 450 nm was measured using a microplate spectrophotometer (Bio-Tek Instruments Inc., Winooski, VT, USA).

### 2.10. Colony Formation Assay

HNSCC cells were placed in 6-well plates (1000 cells/well) after transfection and incubated at 37 °C in DMEM with 10% FBS for 10–14 days. The colonies were washed using PBS and fixed with 4% paraformaldehyde for 15 min. After PBS washing, the colonies were stained with 0.1% crystal violet (Solarbio, Beijing, China) for 15 min and then scanned and counted.

### 2.11. Transwell Assay

Transwell chambers containing 8 µm-pore-size membranes coated with (cell invasion) or without (cell migration) Matrigel (Corning Inc., Corning, NY, USA) were placed in 24-well plates. HNSCC cells were resuspended in 200 µL of serum-free DMEM (1 × 10^5^ cells) and added to the upper chamber. DMEM containing 20% FBS (600 µL) was added to the lower chamber. After 24 h of incubation at 37 °C, cells on the upper side of the membranes were removed using a cotton swab. The migrated or invaded cells were fixed with 4% paraformaldehyde and stained with 0.1% crystal violet (Solarbio). After incubating for 10 min, the membranes were washed using PBS. An optical microscope (Olympus, Tokyo, Japan) was used to acquire images. The number of migrated and invaded cells was randomly counted from three fields and analyzed using ImageJ (NIH, Bethesda, MD, USA).

### 2.12. Tumor Sphere Formation Assay

Ultra-low attachment 6-well plates (Corning Inc.) were used to analyze the tumor sphere formation. HNSCC cells transfected with lentiviruses were plated into the plates. Serum-free DMEM/F12 (Thermo Fisher Scientific, Shanghai, China) medium supplemented with 1% N2 and 1% B27 supplement (Thermo Fisher Scientific) were used to culture the cells. At the same time, 20 ng/mL recombinant human epidermal growth factor (EGF; R&D Systems, Minneapolis, MN, USA) and 10 ng/mL human basic fibroblast growth factor (bFGF; R&D Systems) were also added into the medium [16]. Microscopic images of tumor spheres with diameters greater than 70 μm were acquired after 10 days.

### 2.13. Wound Healing Assay

HNSCC cells were seeded into 6-well plates and transfected. After the cells reached 90–100% confluence, a line wound was scratched on the cell layer using a 200 µL pipette tip and washed with PBS. Cells were incubated in serum-free DMEM at 37 °C for 24 h. Images of the wound were captured under an inverted microscope at 0 and 24 h. Image J (Version 2.9.0) was used to quantify the width of the wound. Wound healing percentage (%) = (wound width at 0 h) − (wound width at 24 h)/(wound width at 0 h) × 100%.

### 2.14. Terminal Deoxynucleotidyl Transferase Nick-End-Labeling (TUNEL) Assay

A TUNEL Apoptosis Assay Kit (Solarbio) was used to detect apoptotic cells following the manufacturer’s instructions. Briefly, cells at 24 h after transfection were incubated with the TUNEL working solution for 1 h at 37 °C. The cells were washed with PBS and fixed with 4% paraformaldehyde for 20 min. The cells were then washed with PBS and stained with DAPI for 10 min. Images were acquired under a fluorescence microscope.

### 2.15. In Vivo Tumor Growth in Mice and Patient-Derived Xenografts (PDXs)

Female BALB/c nude mice (6–8 weeks old) were purchased from SPF Biotechnology Co., Ltd. (Beijing, China). All animal studies strictly conformed to the regulations of the Peking University Biomedical Ethics Committee. For the subcutaneous tumor models, ten mice were randomly divided into two groups (*n* = 5/group). CAL27 cells that were stably transfected with shNC and shMASCC1 were injected subcutaneously (5 × 10^6^ cells/mouse) into the flanks of the mice. Tumor size was measured every week and the weight (g) of the tumors was measured after sacrifice. For the HNSCC PDX models, twenty mice were randomly divided into two groups (*n* = 10/group). PDXs that had been digested into cells were injected (5 × 10^6^ cells/mouse) into the tongues of the mice. After one week, antisense oligonucleotide (ASO) negative control (NC) and ASO MASCC1 (Integrated Biotech Solutions Co.) were injected into the tongues orthotopically and the tail veins of the mice twice a week. The ASO NC sequence was 5′-CCTTCCCTGAAGGTTCCTCC-3′ and the ASO MASCC1 sequence was 5′-AGTATGACTGGGAGACCTCA-3′. For the rescue assays, 28 mice were randomly divided into four groups (*n* = 7/group). CAL27 cells that were stably transfected with NC, miR-195 overexpression lentiviruses, the MASCC1 overexpression vector, or co-transfected with miR-195 overexpression lentiviruses and the MASCC1 overexpression vector were injected (5 × 10^6^ cells/mouse) into the tongues of the mice. Four weeks after inoculation or treatment, the mice were sacrificed, and the samples of tongues and cervical lymph nodes were collected. We calculated the tumor volumes (mm^3^) as V = 1/2 × D × d^2^ (D is the longer diameter; d is the shorter diameter) [16]. Samples were fixed using 4% paraformaldehyde, and embedded in paraffin overnight before they were longitudinally cut and sectioned into 5 µm-thick slices for H&E and immunohistochemistry staining.

### 2.16. Immunostaining

For immunohistochemistry, the prepared 5 µm-thick sections were incubated with the following primary antibodies at 4 °C overnight: anti-Cyclin D1 (ZSGB-BIO, Beijing, China), anti-marker of proliferation Ki67 (Ki67) (1:800; Cell Signaling Technology, Shanghai, China), and anti-pan-cytokeratin (anti-PCK) (Santa Cruz Biotechnology, Shanghai, China). The sections were then incubated with horseradish peroxidase-labeled polymer (ZSGB-BIO) secondary antibody for 1 h at room temperature. Images were acquired under an optical microscope.

### 2.17. Dual-Luciferase Reporter Assay

The miRNA-binding sites on MASCC1 were predicted using RNA22 (https://cm.jefferson.edu/rna22/Interactive/, (accessed on 10 August 2022). Full-length MASCC1 containing the predicted targets for the miR-15/16 family (miR-195/15a/b/16/424/497), chemically modified double-stranded miR-15a/b family mimics, and miRNA mimic controls were designed by and purchased from Integrated Biotech Solutions Co. Briefly, HNSCC cells were grown in a 48-well plate and co-transfected with miRNA mimics or control mimics (100 nM) and the dual luciferase reporter plasmids including wild-type (WT)-MASCC1 and mutated (Mut)-MASCC1 (40 ng/well). Luciferase activity was measured after 24 h of transfection using a Dual-Luciferase Reporter Gene Assay Kit (Beyotime).

### 2.18. Western Blot

HNSCC cells were lysed using radioimmunoprecipitation assay (RIPA) buffer (Solarbio) containing phenylmethylsulfonyl fluoride (PMSF, Beyotime). Protein samples were separated on 10% SDS polyacrylamide gels and transferred to polyvinylidene difluoride (PVDF) membranes. The membranes were blocked using 5% nonfat milk for 1 h and then incubated with primary antibodies at 4 °C overnight. The primary antibodies comprised: anti-Cyclin D1 (1:1000; Cell Signaling Technology), anti-BCL-2 (1:1000; Abmart, Shanghai, China), anti-YAP1 (1:1000; Cell Signaling Technology), and anti-β-actin (1:1000; Cell Signaling Technology). Then, the membranes were incubated with secondary antibodies at room temperature for 1 h. The NcmECL Ultra (NCM Biotech, Suzhou, China) reagent was used to visualize the immunoreactive protein bands. Image J was used to quantify the integrated density of protein bands.

### 2.19. Statistical Analysis

Statistical analyses were carried out using GraphPad Prism 9.5.0 for Windows (GraphPad Software Inc., La Jolla, CA, USA). In vitro experiments were carried out at least three times, and the in vivo experiments were carried out at least twice. Data were expressed as the mean ± standard deviation (SD) or standard error of the mean (SEM). Differences between the two groups were analyzed using Student’s *t*-test. Differences among multiple groups were analyzed by one-way analysis of variance (ANOVA). Survival curves were constructed using the Kaplan–Meier method and were compared using the log-rank test. The correlation between MASCC1 and miR-195 was evaluated using the Pearson test. A two-tailed value of *p* < 0.05 was considered statistically significant.

## 3. Results

### 3.1. MASCC1 Is Highly Expressed in Metastatic HNSCC and Correlates with Patient Prognosis

**Figure 1:**
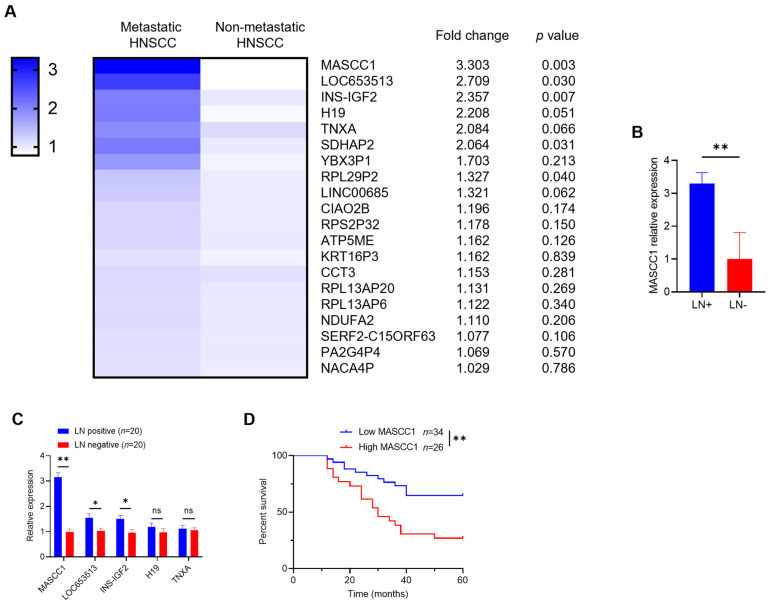
LncRNA MASCC1 is highly expressed in metastatic head and neck squamous cell carcinoma (HNSCC) and correlates with the prognosis of patients with HNSCC. (**A**) The heatmap of the microarray analysis showing the relative expression of different long noncoding RNAs (lncRNAs) in four paired metastatic HNSCC and non-metastatic HNSCC. Fold changes and *p*-values are listed on the right. (**B**) The relative expression of MASCC1 in four paired lymph node-positive groups (LN+) and lymph node-negative groups (LN−) are shown in a histogram. LN+ and LN− represent the metastatic and non-metastatic HNSCC groups, respectively. Values and error bars are shown as the mean ± SD with a paired Student’s *t*-test. (**C**) The relative expression of MASCC1, LOC653513, INS-IGF2, H19, and TNXA in twenty paired LN-positive and LN-negative groups are shown in a histogram. Values and error bars are shown as the mean ± SD with a paired Student’s *t*-test. (**D**) Kaplan–Meier curves with the log-rank test showing the overall survival time of patients with HNSCC with different MASCC1 expression levels (*n* = 60). ns, not significant; * *p* < 0.05 and ** *p* < 0.01.

LncRNA microarray analysis was performed on metastatic HNSCC versus matched non-metastatic HNSCC to identify the differentially expressed lncRNAs. The case–control study was strictly designed to exclude the influence of clinicopathological characteristics, such as sex, age, tumor–node–metastasis (TNM) stage, and pathological differentiation on the lymph node metastasis and prognosis of HNSCC [17]. The four paired samples were matched for sex, age, pathological differentiation, and TNM stage. Using fold change > 3 and *p* < 0.01 as cutoffs, a marked difference in expression was found for MASCC1 between metastatic (lymph node-positive, LN+) and non-metastatic (lymph node-negative, LN−) HNSCC (Figure 1A,B, Table S3). We evaluated the expression of the top five expressed lncRNAs in metastatic HNSCC, MASCC1, LOC653513, INS-IGF2, H19, and TNXA in 20 pairs of metastatic HNSCC and matched non-metastatic HNSCC tissues to validate the microarray analysis findings. The results show that MASCC1 was the most increased lncRNA in metastatic HNSCC versus non-metastatic HNSCC tissues (Figure 1C).

We then analyzed if the MASCC1 expression in HNSCC was correlated with the survival of patients. We set the cut-off point to be the mean fold-change in MASCC1 expression in the 60 patients and split the patients up into high- or low-MASCC1 expression groups. The survival period of the patients with low MASCC1 expression was longer than those with high MASCC1 expression (Figure 1D). Thus, lncRNA MASCC1 seems to affect the prognosis of HNSCC, and its role deserves further investigation. 

### 3.2. Origin Site, Termination Site, Full-length, and Subcellular Localization of MASCC1

**Figure 2:**
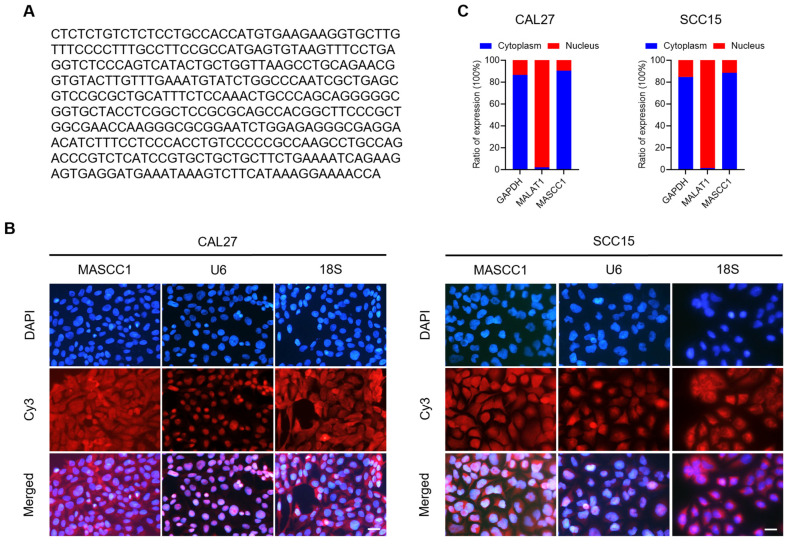
The full-length sequence and subcellular localization of MASCC1. (**A**) The full-length sequence of MASCC1 obtained by means of 5′- and 3′-rapid amplification of cDNA ends (RACE). (**B**) Representative images of MASCC1 cytoplasmic localization provided by the RNA fluorescence in situ hybridization (FISH) assay in CAL27 and SCC15 cells. The nuclei were stained with DAPI. U6 and 18S are markers of the nucleus and cytoplasm, respectively. Scale bar = 20 μm. (**C**) qRT-PCR results show the expression of MASCC1 in the nuclear and cytoplasmic fractions of CAL27 and SCC15. MALAT1 and GAPDH are controls for the nuclear and cytoplasmic fractions, respectively.

We determined whether MASCC1 exists as an intact lncRNA transcript. 5′- and 3′-RACE were used to obtain the full-length MASCC1. 5′-RACE amplified a 183-bp fragment, corresponding to the predicted initiation site for MASCC1 (highlighted in Table S2). The results indicate that the MASCC1 transcript contained a 5′-cap, a typical feature of lncRNAs and messenger RNAs. 3′-RACE identified a 345-bp poly (A)-tailed transcript (highlighted in Table S2). This suggested that the two sequences were not generated from the degraded products of other RNA transcripts. The RACE-obtained full-length sequence of MASCC1 comprised 383 bp (Figure 2A).

Using a Cy3-labeled MASCC1-specific probe, the RNA FISH assay showed that MASCC1 was localized in the cytoplasm of CAL27 and SCC15 cells (Figure 2B). This result was confirmed by means of a nuclear and cytoplasmic fraction isolation assay (Figure 2C). Cytoplasm-located glyceraldehyde-3-phosphate dehydrogenase (GAPDH) [18] and nuclear Metastasis Associated Lung Adenocarcinoma Transcript 1 (MALAT1) [19] were used as controls.

### 3.3. MASCC1 Knockdown Inhibits HNSCC Proliferation, Migration, Invasion, and Tumor Sphere Formation While Promoting HNSCC Apoptosis In Vitro

**Figure 3:**
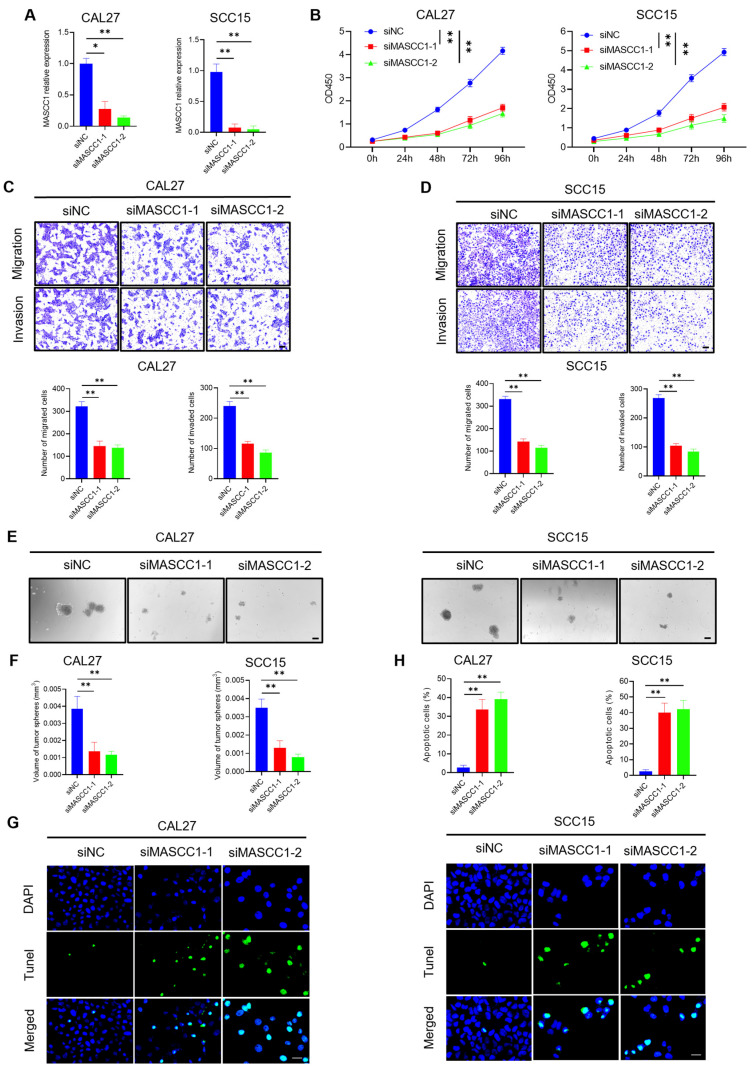
MASCC1 knockdown inhibits HNSCC proliferation, migration, invasion, and tumor sphere formation while promoting HNSCC apoptosis in vitro. (**A**) qRT-PCR showing the relative expression of MASCC1 in CAL27 and SCC15 cells transfected with siNC (short interfering RNA (siRNA) negative control), siMASCC1-1, and siMASCC1-2 after 24 h. Values and error bars are shown as the mean ± SD with an unpaired Student’s *t*-test. (**B**) The effect of MASCC1 knockdown (KD) on the proliferation of CAL27 and SCC15 cells after 0–96 h using the CCK-8 assay. Optical density (OD) 450 values and error bars are shown as the mean ± SD with an unpaired Student’s *t*-test. (**C**) The number of migrated and invaded CAL27 cells with MASCC1 KD using the Transwell assay. Values and error bars are shown as the mean ± SD with an unpaired Student’s *t*-test. Scale bar = 100 μm. (**D**) The number of migrated and invaded SCC15 cells with MASCC1 KD using the Transwell assay. Values and error bars are shown as the mean ± SD with an unpaired Student’s *t*-test. Scale bar = 100 μm. (**E**) Representative images of tumor sphere formation of CAL27 and SCC15 cells with MASCC1 KD. Scale bar = 100 μm. (**F**) The volume of tumor spheres formed by CAL27 and SCC15 cells with MASCC1 KD. Values and error bars are shown as the mean ± SD with an unpaired Student’s *t*-test. (**G**) Representative images of the TUNEL assay showing the apoptotic CAL27 and SCC15 cells with MASCC1 KD. Scale bar = 20 μm. (**H**) The apoptosis rate of CAL27 and SCC15 with MASCC1 KD. Values and error bars are shown as the mean ± SD with an unpaired Student’s *t*-test. * *p* < 0.05 and ** *p* < 0.01.

To understand the biological functions of MASCC1 in HNSCC, small interfering RNAs (siRNAs), including siNC, siMASCC1-1, and siMASCC1-2, were transfected into CAL27 and SCC15 cells. The results show that MASCC1 KD by siMASCC1-1/2 reduced the expression of MASCC1 at 24 h after transfection (Figure 3A). CCK-8 and colony formation assays indicated that MASCC1 KD inhibited CAL27 and SCC15 cell proliferation (Figure 3B and Figure S1A). Transwell assays demonstrated that MASCC1 KD suppressed CAL27 and SCC15 cell migration and invasion (Figure 3C,D). The wound healing assay also confirmed that MASCC1 KD inhibited HNSCC cell migration (Figure S1B). MASCC1 KD inhibited the tumor sphere formation of CAL27 and SCC15 cells compared with the siNC group (Figure 3E,F). The TUNEL assay revealed that MASCC1 KD promoted CAL27 and SCC15 cell apoptosis (Figure 3G,H).

### 3.4. MASCC1 Knockdown Suppresses Tumor Formation and Lymph Node Metastasis In Vivo

**Figure 4:**
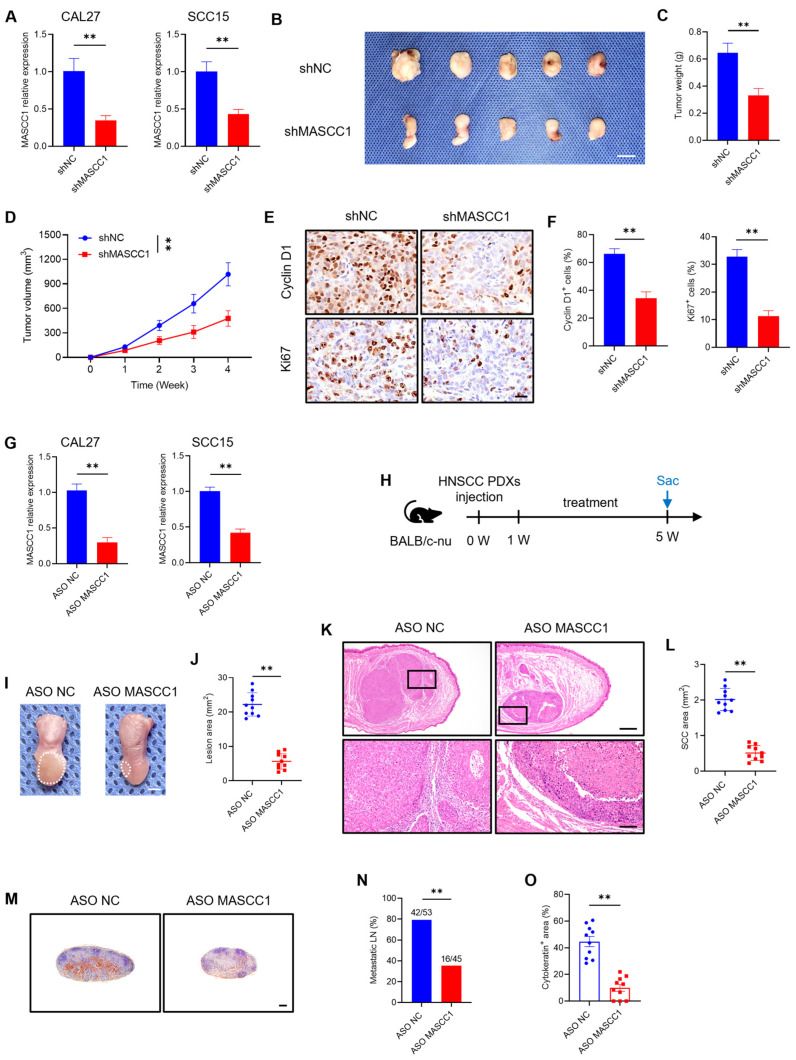
MASCC1 KD inhibits HNSCC growth and lymph node metastasis in vivo. (**A**) qRT-PCR analysis showing the relative expression of MASCC1 in CAL27 and SCC15 cells transfected with shNC (short hairpin RNA (shRNA) negative control) and shMASCC1 after 48 h. Values and error bars are shown as mean ± SD with an unpaired Student’s *t*-test. (**B**) Image of subcutaneous tumors formed by CAL27 cells transfected with shNC and shMASCC1 in nude mice. Scale bar = 1 cm. (**C**) Tumor weight of subcutaneous tumors formed by CAL27 cells with MASCC1 knockdown (KD) in nude mice. Values and error bars are shown as mean ± SD with an unpaired Student’s *t*-test (*n* = 5). (**D**) Tumor volume growth curves of subcutaneous tumors formed by CAL27 cells with MASCC1 KD in nude mice for four weeks. Values and error bars are shown as mean ± SD with an unpaired Student’s *t*-test (*n* = 5). (**E**) Immunohistochemistry (IHC) images showing the expression of Cyclin D1 and Ki-67 in subcutaneous tumors of nude mice formed by CAL27 cells with MASCC1 KD. Scale bar = 20 μm. (**F**) The expression percentage of Cyclin D1 and Ki67 in subcutaneous tumors of the different groups. Values and error bars are shown as the mean ± SD with an unpaired Student’s *t*-test. (**G**) qRT-PCR analysis showing the relative expression of MASCC1 in CAL27 and SCC15 cells transfected with antisense oligonucleotide negative control (ASO NC) and ASO MASCC1 after 24 h. Values and error bars are shown as the mean ± SD with an unpaired Student’s *t*-test. (**H**) The schematic diagram showing the timeline of mice injected with HNSCC patient-derived xenografts (PDXs), antisense oligonucleotide (ASO) MASCC1 treatment, and sacrifice (SAC). (**I**) Representative images of PDX orthotopic tongue lesions from nude mice in the different groups. White dashed circles mark the lesion areas. Scale bar = 2 mm. (**J**) Quantification of PDX lesion areas from nude mice in the different groups as indicated. Values and error bars are shown as the mean ± SD with an unpaired Student’s *t*-test (*n* = 10). (**K**) Representative hematoxylin and eosin (H&E) staining images of PDX orthotopic tongue tumors from nude mice in different groups. Scale bar = 500 μm. Magnified images are shown in the lower panels. Scale bar = 100 μm. (**L**) Quantification of the squamous cell carcinoma (SCC) area from nude mice in the different groups as indicated. Values and error bars are shown as the mean ± SD with an unpaired Student’s *t*-test (*n* = 10). (**M**) Representative immunostaining images of metastatic cells in cervical lymph nodes by anti-pan-cytokeratin (PCK) from the different groups. Scale bar = 200 μm. (**N**) The percentage of metastatic lymph nodes from nude mice in different groups. The number of metastatic lymph nodes (LN) is indicated in the histogram. The result represents the average of two independent experiments by the Chi-squared test. (**O**) Quantification of the metastatic area in cervical lymph nodes from nude mice in the different groups. Values and error bars are shown as the mean ± SEM from two independent experiments with an unpaired Student’s *t*-test. ** *p* < 0.01.

To explore whether MASCC1 KD inhibited tumor growth in vivo, shMASCC1 was used to knock down MASCC1. qRT-PCR showed that MASCC1 was reduced at 48 h after transfection with shMASCC1 in CAL27 and SCC15 cells (Figure 4A). In vivo, at four weeks after the injection of MASCC1 KD CAL27 cells, the subcutaneous tumors grown in nude mice were smaller than those in the shNC group (Figure 4B). Consistently, MASCC1 KD also reduced the weight and volume of the subcutaneous tumors in vivo (Figure 4C,D). Immunostaining tested the expression of Cyclin D1 (cell cycle marker [20]) and Ki67 (cell proliferation marker [21]) in the subcutaneous tumors of nude mice (Figure 4E). MASCC1 KD by shMASCC1 reduced the percentage of Cyclin D1 and Ki67 expression in the subcutaneous tumors of nude mice (Figure 4F).

ASOs can knock down genes associated with cancer and effectively inhibit tumor growth in vitro and in vivo, thus providing prospects for the clinical translation of HNSCC treatment [22,23,24]. qRT-PCR analysis showed that ASO MASCC1 effectively knocked down the expression of MASCC1 in CAL27 and SCC15 cells (Figure 4G). We used ASO MASCC1 to further investigate whether MASCC1 KD could suppress orthotopic PDXs of HNSCC tumor growth and lymph node metastasis in nude mice. One week after injection with HNSCC PDX cells, mice were randomly divided into two groups and treated with ASO NC or ASO MASCC1 by means of intra-tumoral and tail intravenous injection twice a week, for four weeks (Figure 4H). Compared with that in the ASO NC group, tumor growth was inhibited in the ASO MASCC1 group (Figure 4I–L). Moreover, immunostaining of anti-PCK showed that cervical lymph node metastasis was suppressed in the ASO MASCC1 group compared with that in the ASO NC group (Figure 4M–O). These results demonstrate that MASCC1 could serve as an efficient therapeutic target for HNSCC treatment.

### 3.5. MASCC1 Acts as a Competing Endogenous RNA (ceRNA) by Sponging miR-195, Thus Regulating Cyclin D1, BCL-2, and YAP1 Expression

**Figure 5:**
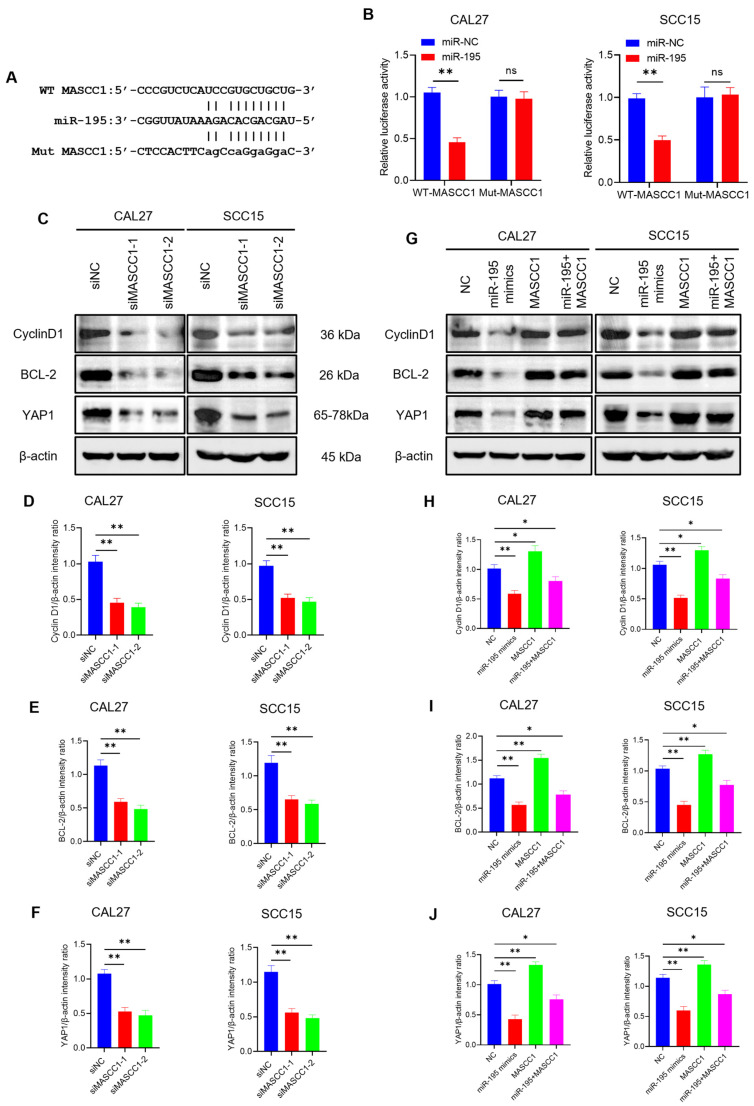
MASCC1 functions as a sponge for miR-195 in HNSCC cells. (**A**) The seed sequence of the miR-195 (**middle**), matches the binding sites of MASCC1 (WT; (**upper**)), along with mutations of the binding sites of MASCC1 (MUT; (**lower**)). (**B**) Histograms showing the effects of miR-195 on CAL27 and SCC15 cells with wild-type or mutated binding sites of MASCC1, reported as firefly luciferase activity. Values and error bars are shown as the mean ± SD with an unpaired Student’s *t*-test. (**C**) Western blot analysis showing the protein levels of Cyclin D1, BCL-2, and YAP1 in CAL27 and SCC15 cells transfected with small interfering RNAs (siRNAs). β-actin was used as the internal control. The molecular weights of the proteins are listed on the right. The uncropped blots are shown in Supplementary File S1. (**D**–**F**) Diagrams showing the intensity ratio of Cyclin D1/β-actin, BCL-2/β-actin, and YAP1/β-actin in CAL27 and SCC15 cells with MASCC1 KD. Values and error bars are shown as the mean ± SD with an unpaired Student’s *t*-test. (**G**) Western blot analysis showing the relative protein levels of Cyclin D1, BCL-2, and YAP1 in CAL27 and SCC15 cells transfected with the negative control (NC), miR-195 mimics, and pcDNA3.1-MASCC1, or co-transfected with miR-195 mimics and pcDNA3.1-MASCC1. β-actin was used as the internal control. The uncropped blots are shown in Supplementary File S1. (**H**–**J**) Diagrams showing the intensity ratio of Cyclin D1/β-actin, BCL-2/β-actin, and YAP1/β-actin in CAL27 and SCC15 cells transfected with the negative control (NC), miR-195 mimics, and MASCC1, or co-transfected with miR-195 mimics and MASCC1. Values and error bars are shown as the mean ± SD by one-way analysis of variance (ANOVA). ns, not significant; * *p* < 0.05 and ** *p* < 0.01.

LncRNAs can bind target microRNAs (miRNAs) and regulate the expression of their target genes, functioning as ceRNAs [25,26,27]. Given that MASCC1 is mainly located in the cytoplasm, we hypothesized that MASCC1 binds to miRNAs to regulate target mRNAs. RNA22 predicted that MASCC1 contains target sites for the miR-15/16 family (Figure 5A and Figure S3A,D). Thus, we constructed luciferase reporter plasmids containing the wild-type (WT) and mutant target (Mut) sites in the MASCC1 sequence. CAL27 and SCC15 cells were co-transfected with miRNA mimics or miR-NC mimics and reporter plasmids. 

Ectopic miR-195 expression suppressed the relative firefly luciferase activity of the WT MASCC1 reporter, but not that of the Mut MASCC1 reporter (Figure 5B), indicating that miR-195 binds to MASCC1. The relative firefly luciferase activity did not change in CAL27 and SCC15 cells co-transfected with miR-15a/15b or miR-16/424/497 and WT MASCC1 or Mut MASCC1 (Figure S3B,C and Figure S3E–G, respectively). Thus, we chose miR-195 for further study.

To explore whether MASCC1 regulates the expression of genes associated with miR-195, we measured the protein levels of Cyclin D1, BCL-2, and YAP1 using Western blots. Cyclin D1, BCL-2, and YAP1 levels are associated with the cell cycle [28], apoptosis [29], and metastasis [30], respectively. siMASCC1-1/2-mediated MASCC1 KD reduced the protein levels of Cyclin D1, BCL-2, and YAP1 in CAL27 and SCC15 cells (Figure 5C–F). qRT-PCR confirmed that the expression levels of the three mRNAs were reduced after transfection with siMASCC1-1/2 in CAL27 and SCC15 cells (Figure S4A–C). 

Collectively, MASCC1 functions as a ceRNA that directly binds to miR-195 to regulate Cyclin D1, BCL-2, and YAP1 expression.

### 3.6. miR-195 Overexpression Reverses the Effects of MASCC1 Overexpression on the Biological Behaviors of HNSCC In Vitro and In Vivo

The above results suggest that miR-195 is involved in the progression and metastasis of HNSCC by interacting with MASCC1. Thus, we performed rescue assays to explore whether miR-195 could reverse the effects of MASCC1 on the biological behaviors of HNSCC.

**Figure 6:**
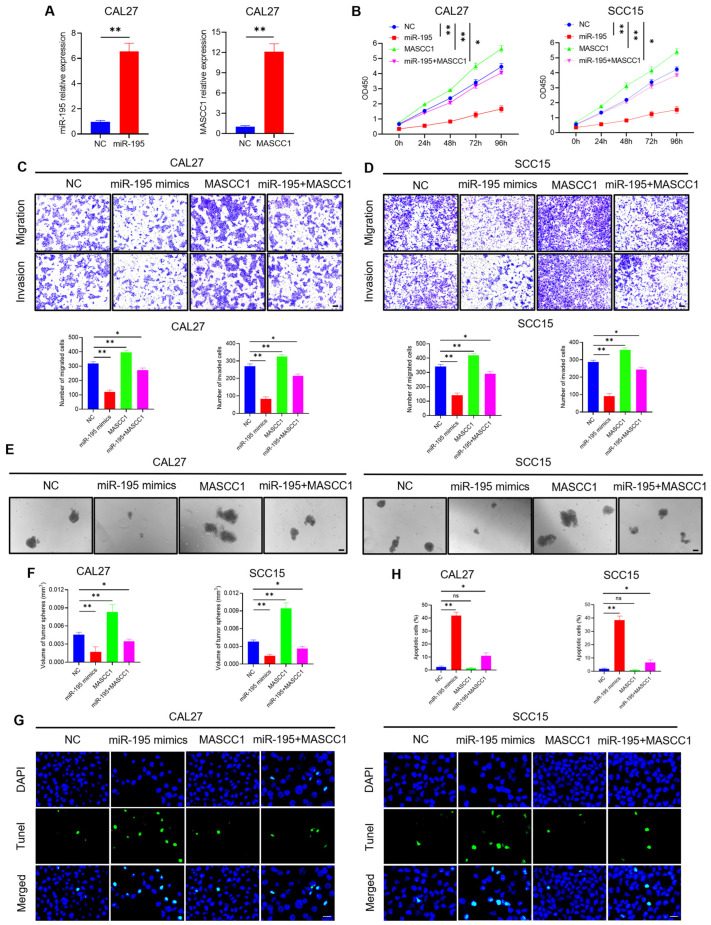
miR-195 overexpression rescues the effects of MASCC1 overexpression on HNSCC in vitro. (**A**) qRT-PCR analysis showing the relative expression of miR-195 and MASCC1 in CAL27 cells transfected with negative control (NC), miR-195 mimics, and MASCC1 after 24 h. Values and error bars are shown as the mean ± SD with an unpaired Student’s *t*-test. (**B**) The effect of transfection with NC, miR-195 mimics, and MASCC1, or co-transfected with miR-195 mimics and MASCC1 on the proliferation of CAL27 and SCC15 after 0–96 h using the CCK-8 assay. Optical density (OD) 450 values and error bars are shown as the mean ± SD by one-way analysis of variance (ANOVA). (**C**) The number of migrated and invaded CAL27 cells transfected with NC, miR-195 mimics, and MASCC1, or co-transfected with miR-195 mimics and MASCC1 using the Transwell assay. Values and error bars are shown as the mean ± SD by one-way ANOVA. Scale bar = 100 μm. (**D**) The number of migrated and invaded SCC15 cells transfected with NC, miR-195 mimics, and MASCC1, or co-transfected with miR-195 mimics and MASCC1 using the Transwell assay. Values and error bars are shown as the mean ± SD by one-way ANOVA. Scale bar = 100 μm. (**E**) Representative images of tumor sphere formation of CAL27 and SCC15 cells transfected with NC, miR-195 mimics, and MASCC1, or co-transfected with miR-195 mimics and MASCC1. Scale bar = 100 μm. (**F**) The volume of tumor spheres formed by CAL27 and SCC15 cells transfected with NC, miR-195 mimics, and MASCC1, or co-transfected with miR-195 mimics and MASCC1. Values and error bars are shown as the mean ± SD by one-way ANOVA. (**G**) Representative images of TUNEL assays showing the apoptotic CAL27 and SCC15 cells transfected with NC, miR-195 mimics, and MASCC1, or co-transfected with miR 195 mimics and MASCC1. Scale bar = 20 μm. (**H**) The apoptosis rate of CAL27 and SCC15 cells transfected with NC, miR-195 mimics, and MASCC1, or co-transfected with miR-195 mimics and MASCC1. Values and error bars are shown as mean ± SD by one-way ANOVA. ns, not significant, * *p* < 0.05, and ** *p* < 0.01.

CAL27 and SCC15 cells were transfected with NC, miR-195 mimics, pcDNA3.1-MASCC1, or co-transfected with miR-195 mimics and MASCC1. The results show that the relative expression of miR-195 and MASCC1 increased after transfection with miR-195 mimics and MASCC1 in CAL27 and SCC15 compared with that in the NC group (Figure 6A and Figure S2A). Western blots indicated that MASCC1 overexpression enhanced the Cyclin D1, BCL-2, and YAP1 levels. However, miR-195 overexpression reduced the levels of these proteins. miR-195 overexpression reversed MASCC1 overexpression’s effect on protein levels (Figure 5G–J). qRT-PCR analysis demonstrated that the mRNA expression of Cyclin D1, BCL-2, and YAP1 was consistent with the results of the Western blot analysis (Figure S4D–F).

In vitro, MASCC1 overexpression promoted the proliferation, migration, invasion, and tumor sphere formation of CAL27 and SCC15 cells, while inhibiting their apoptosis (Figure 6B–H and Figure S2B–E), whereas miR-195 overexpression had the opposite effects (Figure 6B–H and Figure S2B–E). Thus, miR-195 overexpression reversed the effects of MASCC1 overexpression on HNSSC biological behaviors.

**Figure 7:**
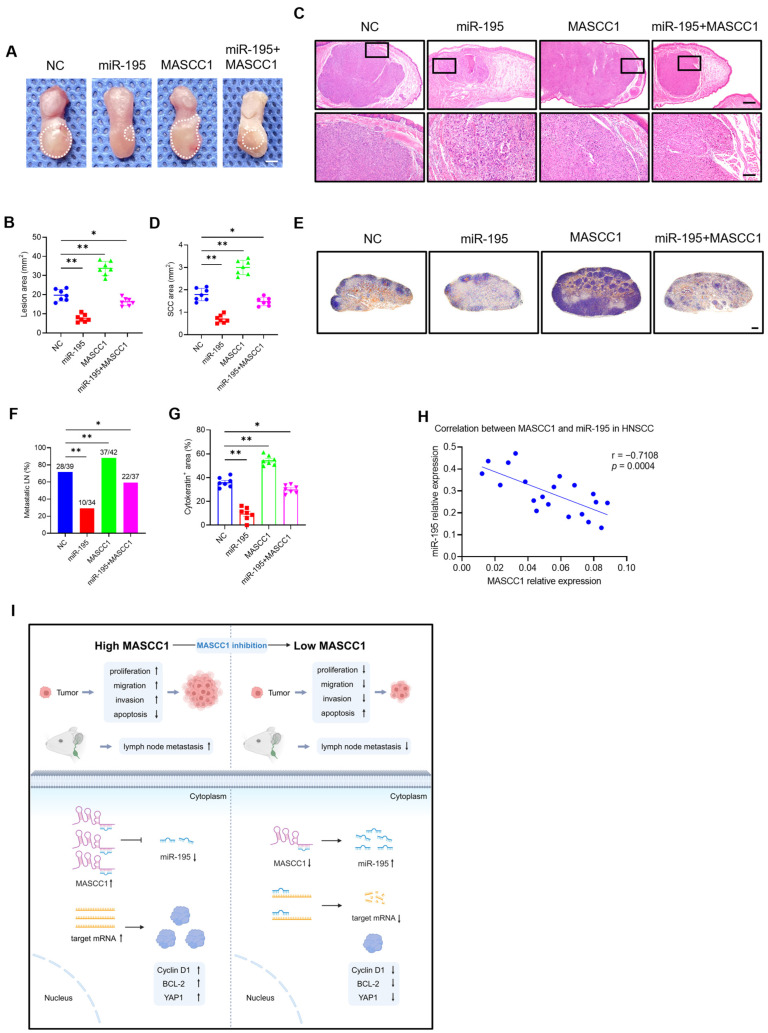
miR-195 overexpression rescues the effects of MASCC1 overexpression on HNSCC tumor growth and lymph node metastasis in vivo. (**A**) Representative images of HNSCC orthotopic tongue lesions from nude mice in the different groups. White dashed circles mark the lesion areas. Scale bar = 2 mm. (**B**) Quantification of the HNSCC lesion area from nude mice in the different groups as indicated. Values and error bars are shown as the mean ± SD by one-way ANOVA (*n* = 7). (**C**) Representative hematoxylin and eosin (H&E) staining images of HNSCC orthotopic tongue tumors from nude mice in the different groups. Scale bar = 500 μm. Magnified images are shown in the lower panels. Scale bar = 100 μm. (**D**) Quantification of the squamous cell carcinoma (SCC) area from nude mice in the different groups as indicated. Values and error bars are shown as the mean ± SD by one-way ANOVA (*n* = 7). (**E**) Representative immunostaining images of metastatic cells in cervical lymph nodes using anti-pan-cytokeratin (PCK) from the different groups. Scale bar = 200 μm. (**F**) The percentage of metastatic lymph nodes (LN) from nude mice in the different groups. The number of metastatic lymph nodes is indicated in the histogram. The result represents the average of two independent experiments by the Chi-squared test. (**G**) Quantification of the metastatic area in cervical lymph nodes from nude mice in the different groups. Values and error bars are shown as the mean ± SEM from two independent experiments by one-way ANOVA. (**H**) Correlation between the relative expression of MASCC1 and miR-195 in HNSCC tumor tissues (*n* = 20). The values of r and *p* from the Pearson test are presented on the right. (**I**) A schematic diagram (created with Biorender.com) illustrating the mechanism by which MASCC1 sponges miR-195 and regulates the progression and metastasis of HNSCC. * *p* <0.05 and ** *p* < 0.01.

We performed rescue experiments in vivo. After CAL27 cells were stably transfected with NC, miR-195 overexpression lentiviruses, and MASCC1 overexpression vectors, or co-transfected with miR-195 overexpression lentiviruses and MASCC1 overexpression vector, they were injected into the tongues of nude mice. In vivo, MASCC1 overexpression promoted HNSCC tumor formation and lymph node metastasis in nude mice compared with those in the NC group (Figure 7A–G). In contrast, miR-195 overexpression inhibited HNSCC tumor formation and lymph node metastasis in nude mice, thus alleviating MASCC1-induced carcinogenesis (Figure 7A–G).

We evaluated the relative expression of MASCC1 and miR-195 in tumor tissues from 20 patients with HNSCC. The relative expression of miR-195 correlated negatively with MASCC1 expression in these HNSCC samples (Figure 7H). 

Thus, miR-195 overexpression rescued the effects of MASCC1 overexpression on HNSCC progression and metastasis in vitro and in vivo. 

## 4. Discussion

LncRNAs are reported to be closely related to tumor development and metastasis [31,32,33]. HNSCC progression is a multi-step and heterogeneous process involving various genetic and epigenetic changes [6,34,35]. LncRNAs have a wide range of biological functions and are thus involved in HNSCC carcinogenesis, progression, and metastasis, e.g., HOTAIR [36], lncMX1-215 [37], and MALAT1 [38]. Here, we identified a novel lncRNA, *MASCC1*, which was highly expressed in metastatic HNSCC and was correlated with poor prognosis. We focused on MASCC1 since it had one of the highest fold changes among the lncRNAs dysregulated in HNSCC and its functional effects have never been reported. RACE, FISH, and qRT-PCR assays confirmed the presence and aberrant expression of MASCC1 in HNSCC. In vitro, MASCC1 KD inhibited proliferation, migration, invasion, and tumor sphere formation while promoting apoptosis. In vivo, MASCC1 KD suppressed HNSCC tumor formation and lymph node metastasis. Mechanistically, MASCC1 acted as a ceRNA to sponge miR-195, thereby regulating Cyclin D1, BCL-2, and YAP1 expression (Figure 7I). Our results indicate that lncRNA *MASCC1* functions as an oncogene and is involved in HNSCC progression, tumorigenesis, and metastasis, making it a potential target for HNSCC treatment. 

ASOs have been applied to inhibit the development and metastasis of lung cancer [22], breast cancer [24], and colon cancer [39], thus showing great potential for clinical translation and application in cancer therapy [40]. PDXs have been used as preclinical models because of their genetic similarity to human cancers [41]. Here, HNSCC PDX-injected nude mice confirmed the therapeutic effect of ASO MASCC1, i.e., the inhibition of tumor formation and lymph node metastasis of HNSCC. These results suggest MASCC1 as an effective target for HNSCC therapy and the value of ASO MASCC1 for clinical translation in HNSCC treatment.

LncRNAs have diverse regulatory mechanisms in human diseases. In addition to chromatin modification, transcription, and post-transcriptional processing, lncRNAs can serve as ceRNAs or miRNA inhibitors to regulate miRNAs and the expression of their target genes [8,42,43]. Several lncRNAs regulate HNSCC by functioning as ceRNAs, including RC3H2 [44], AC104041.1 [45], and LINC00520 [46]. Here, we found that MASCC1 played an oncogenic role, largely acting as a ceRNA for miR-195, thereby regulating Cyclin D1, BCL-2, and YAP1 expression. miR-195 is involved in the regulation of cancer by targeting Cyclin D1 [47], BCL-2 [48], and YAP1 [49]. Cyclin D1 is a key protein participating in cell cycle control and is essential for the G1 to S transition [28]. BCL-2 is a key regulator of apoptosis and confers a survival advantage on cells by protecting them from apoptotic death [50]. The ectopic expression of YAP1 is associated with lymph node metastasis [51]. Previously, we showed an anti-tumor effect of miR-195 in HNSCC via the inhibition of Cyclin D1 and BCL-2 expression [52]. Thus, these findings indicate that MASCC1 might modulate HNSCC by promoting the cell cycle, cell apoptosis, and metastasis.

In addition, researchers have conducted rescue assays to explore whether the biological functions of lncRNAs could be reversed by miRNAs in cancers [53,54,55]. Here, rescue assays demonstrated that MASCC1 overexpression promoted HNSCC cell proliferation, migration, invasion, tumor sphere formation, and lymph node metastasis while suppressing apoptosis, rescued by miR-195 overexpression. Moreover, MASCC1 and miR-195 expression correlated negatively in HNSCC samples. Collectively, these results suggest that MASCC1 regulates HNSCC progression and metastasis by interacting with miR-195.

## 5. Conclusions

Our results demonstrate that a novel lncRNA, *MASCC1*, acts as an oncogene and regulates the progression and metastasis of HNSCC by sponging miR-195. These findings provide a better understanding of the pathogenesis, progression, and metastasis of HNSCC, and suggest that lncRNA MASCC1 has potential value as a therapeutic target in patients with HNSCC.

## Data Availability

The data presented in this study are available from the corresponding author upon reasonable request. The raw and processed microarray data were deposited in the Gene Expression Omnibus database in the National Center for Biotechnology Information with the accession number GSE246104.

## Supplementary Materials

The following supporting information can be downloaded at: https://www.mdpi.com/article/10.3390/cancers15245792/s1, Figure S1: MASCC1 knockdown inhibits HNSCC proliferation and migration in vitro; Figure S2: miR-195 overexpression rescues the effects of MASCC1 overexpression on HNSCC proliferation and migration in vitro; Figure S3: The miR-15/16 family binding sites on MASCC1; Figure S4: MASCC1 regulates the expression of Cyclin D1, BCL-2, and YAP1 mRNAs in HNSCC; Supplementary File S1: The uncropped blots of Figure 5C,G. Table S1: Sequences of qRT-PCR primers used in the study; Table S2: Sequences of 5′- and 3′-Rapid Amplification of cDNA Ends (RACE); Table S3: The relative expression of different lncRNAs in four paired metastatic head and neck squamous cell carcinoma (HNSCC) and non-metastatic HNSCC samples using microarray analysis.
