# Supplementary material for: A Novel LncRNA MASCC1 Regulates the Progression and Metastasis of Head and Neck Squamous Cell Carcinoma by Sponging miR-195
Source: Cancers (Basel). 2023 Dec 11;15(24):5792. doi: 10.3390/cancers15245792 (PMC10741893; doi:10.3390/cancers15245792)
Supplement: Supplementary file 1 [file cancers-15-05792-s001.zip › Supplementary Tables and Figures.pdf]

Table S1. Sequences of qRT-PCR primers used in the study.

Target genes	Forward (5'-3')	Reverse (5'-3')
MASCC1	GTATCTGGCCCAATCGCTGA	AGGAAAGATGTTCCTCGCCC
LOC653513	CCAGGAAAGAGTGTCTGTGG	ATGTCTCTTGCTTGCCGCTA
INS-IGF2	ATCATCGTCCAGGCAGTTTCGG	ACACAAGCTCGGTGGTGACTCT
H19	CTTGGAAATGAATATGCTGCAC	TTCCTCTAGCTTCACCTTCC
TNXA	CGTGGTCCAGTATGAGGACAC	TAGAGGAGGAACCTGTAGGGG
GAPDH	GGAGCGAGATCCCTCCAAAAT	GGCTGTTGTCATACTTCTCATGG
MALAT1	GTTCTGATCCCGCTGCTATT	TCCTCAACACTCAGCCTTTATC
miR-195	AGCTTCCCTGGCTCTAGCA	CTGGAGCAGCACAGCCAATA
Cyclin D1	CAATGACCCCGCACGATTTC	CATGGAGGGCGGATTGGAA
BCL-2	GGTGGGGTCATGTGTGTGG	CGGTTCAGGTACTCAGTCATCC
YAP1	TAGCCCTGCGTAGCCAGTTA	TCATGCTTAGTCCACTGTCTGT

Table S2. Sequences of 5′- and 3′-Rapid Amplification of cDNA Ends (RACE).

	Sequences
5R-nestA	TGCTGGGCAGTTTGGAGAA
5R-nestB	TCAGCGATTGGGCCAGATA
5′ RACE	TCCCCGCGCGTTGGCCGATTCATTAATGCAGCTGGCACGACAGGTTTCCCGA<br>CTGGAAAGCGGGCAGTGAGCGCAACGCAATTAATGTGAGTTAGCTCACTCAT<br>TAGGCACCCCAGGCTTTACACTTTATGCTTCCGGCTCGTATGTTGTGTGGAAT<br>TGTGAGCGGATAACAATTTCACACAGGAAACAGCTATGACCATGATTACGCC<br>AAGCGCGCAATTAACCCTCACTAAAGGGAACAAAAGCTGGAGCTCCACCGC<br>GGTGGCGGCCGCTCTAGAACTAGTGGATCCCCCGGGCTGCAGGAATTCCCAA<br>TACT**AAGCAGTGGTATCAACGCAGAGT**ACATGGGCTCTCTGTCTCTCCTGC<br>CACCATGTGAAGAAGGTGCTTGTTTCCCCTTTGCCTTCCGCCATGAGTGTAA<br>GTTTCCTGAGGTCTCCCAGTCATACTGCTGGTTAAGCCTGCAGAACGGTGTAC<br>TTGTTTGAAATG**TATCTGGCCCAATCGCTGA**AGTATTGGGAATTCGATATCA<br>AGCTTATCGATACCGTCGACTCGAGGGACCCAGCCAGCCT
3′ RACE	GGGATGAGATCCATGGGCGGCGCCTGCAGACCAGGTCT**CTCCCAGTCATAC**<br>**TGCTGGTTAA**GCCTGCAGAACGGTGTACTTGTTTGAAATGTATCTGGCCCAA<br>TCGCTGAGCGTCCGCGCTGCATTTCTCCAAACTGCCCAGCAGGGGGCGGTGC<br>TACCTCGGCTCCGCGCAGCCACGGCTTCCCGCTGGCGAACCAAGGGCGCGG<br>AATCTGGAGAGGGCGAGGAACATCTTTCCTCCCACCTGTCCCCCGCCAAGCC<br>TGCCAGACCCGTCTCATCCGTGCTGCTGCTTCTGAAAATCAGAAGAGTGAGG<br>ATGAAATAAAGTCTTCATAAAGGAAAACCAAAAAAAAAAA**CCTATAGTGAA**<br>**ATCACTAGTGGAACGACGGTAA**AGACTGGAGATCTGGATCCCTCGAGTCTA<br>GAGTCGACCTGCAGGCATGCAAGCTTGGCGTAATCATGGTCATAGCTGTTTCC<br>TGTGTGAAATTGTTATCCGCTCACAATTCCACACAACATACGAGCCGGAAGCA<br>TAAAGTGTAAAGCCTGGGGTGCCTAATGAGTGAGCTAACTCACATTAATTGC<br>GTTGCGCTCACTGCCCGCTTTCCAGTCGGGAAACCTGTCGTGCCAGCTGCAT<br>TAATGAATCGGCCAACGCGCGGGGAGAGGCGGTTTGCGTATTGGGCGCTCTT<br>CCGCTTCCTCGCTCACTGACTCGCTGCGCTCGGTC

Table S3. The relative expression of different lncRNAs in four paired metastatic head and neck squamous cell carcinoma (HNSCC) and non-metastatic HNSCC samples using microarray analysis.

	1LN+	2LN+	3LN+	4LN+	5LN-	6LN-	7LN-	8LN-	Fold change	P value
MASCC1	3.664	3.457	3.170	2.921	2.121	1.013	0.573	0.293	3.303	0.003
LOC653513	2.609	2.587	2.873	2.766	2.124	0.920	0.627	0.328	2.709	0.030
INS-IGF2	3.366	1.945	2.079	2.038	1.670	0.801	1.200	0.328	2.357	0.007
H19	1.847	1.781	2.316	2.887	1.603	0.799	0.714	0.884	2.208	0.051
TNXA	3.107	1.398	1.962	1.870	1.347	0.907	1.608	0.139	2.084	0.066
SDHAP2	2.023	2.162	1.687	2.386	1.456	0.990	0.964	0.590	2.064	0.031
YBX3P1	1.132	1.641	1.954	2.087	1.683	0.848	0.923	0.547	1.703	0.213
RPL29P2	1.227	1.429	1.410	1.244	1.145	0.946	0.946	0.963	1.327	0.040
LINC00685	1.325	1.116	1.249	1.597	1.122	0.934	0.996	0.948	1.321	0.062
CIAO2B	1.130	1.233	1.081	1.342	1.198	0.878	0.984	0.940	1.196	0.174
RPS2P32	1.125	1.165	1.155	1.268	1.177	0.915	1.026	0.883	1.178	0.150
ATP5ME	1.175	1.174	0.918	1.382	1.100	0.948	0.916	1.036	1.162	0.126
KRT16P3	0.435	2.073	1.420	0.719	1.862	0.402	0.324	1.412	1.162	0.839
CCT3	0.951	1.382	1.092	1.185	1.046	1.088	1.081	0.785	1.153	0.281
RPL13AP20	1.134	1.047	1.131	1.211	1.181	0.751	0.832	1.236	1.131	0.269
RPL13AP6	1.059	1.003	1.256	1.172	1.133	0.824	0.853	1.191	1.122	0.340
NDUFA2	1.222	1.086	0.953	1.180	1.134	1.061	0.935	0.870	1.110	0.206
SERF2-C15ORF63	1.149	0.977	1.097	1.084	1.148	0.923	1.004	0.924	1.077	0.106
PA2G4P4	0.988	1.093	1.132	1.064	1.173	0.841	0.884	1.102	1.069	0.570
NACA4P	1.058	1.071	0.878	1.107	1.251	0.967	0.927	0.854	1.029	0.786

Note: Lymph node-positive (LN+), lymph node-negative (LN-).

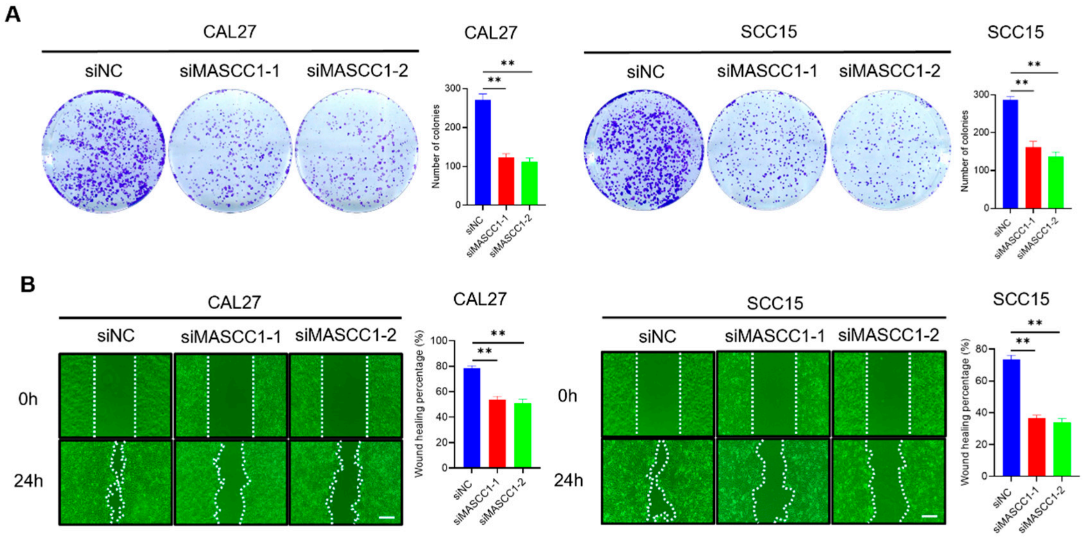

Figure S1. MASCC1 knockdown inhibits HNSCC proliferation and migration in vitro. (**A**) The colony numbers of CAL27 and SCC15 cells with MASCC1 knockdown (KD) according to colony formation assays. Values and error bars are shown as the mean ± SD with an unpaired Student's t-test. (**B**) Representative images and percentage of wound healing showing the migration of CAL27 and SCC15 cells with MASCC1 KD. Values and error bars are shown as the mean ± SD with an unpaired Student's t-test. Scale bar = 100 μm. ** $p < 0.01$.

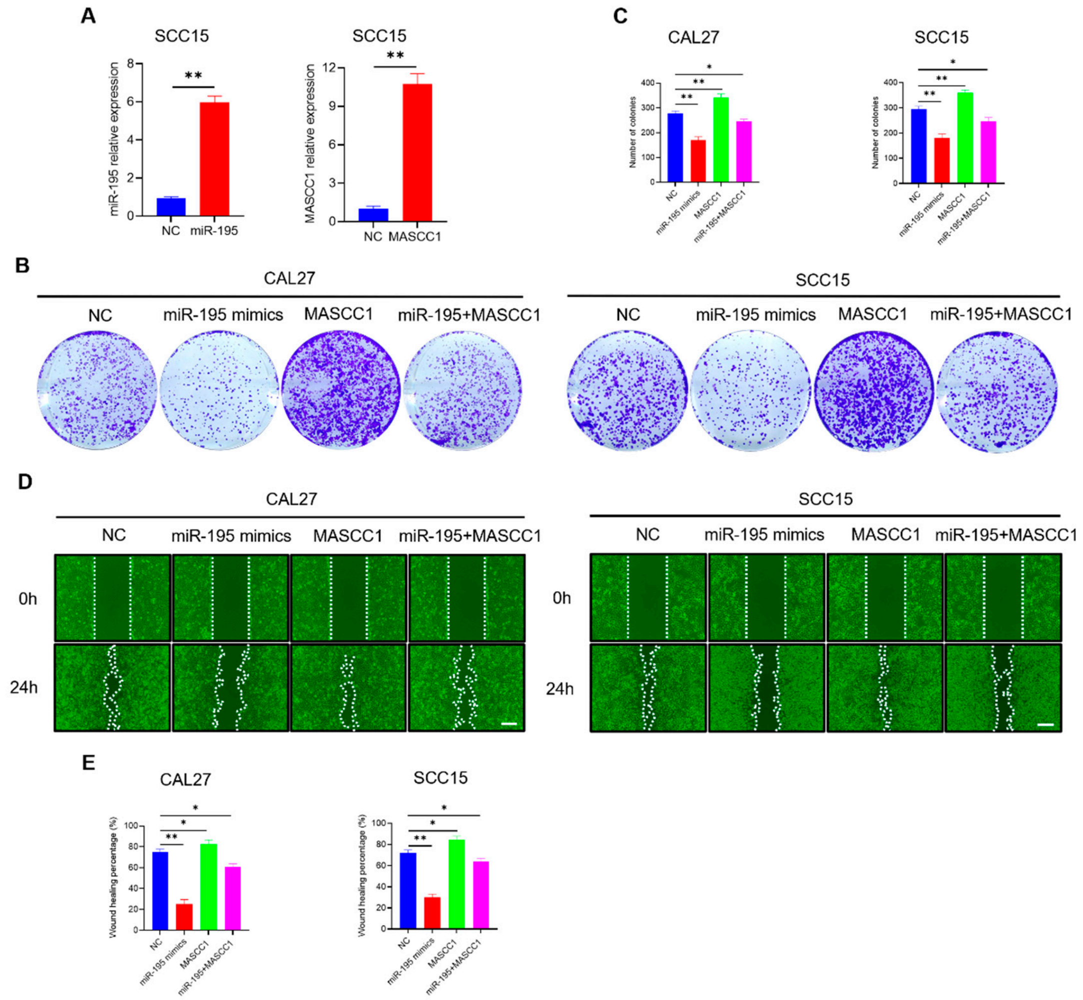

Figure S2. miR-195 overexpression rescues the effects of MASCC1 overexpression on HNSCC proliferation and migration in vitro. (**A**) qRT-PCR analysis showing the relative expression of miR-195 and MASCC1 in SCC15 cells transfected with negative control (NC), miR-195 mimics, and MASCC1 after 24 h. Values and error bars are shown as the mean ± SD with an unpaired Student's t-test. (**B**) The colony formation of CAL27 and SCC15 cells transfected with NC, miR-195 mimics, MASCC1, or co-transfected with miR-195 mimics and MASCC1. (**C**) The colony numbers of CAL27 and SCC15 cells transfected with NC, miR-195 mimics, and MASCC1, or co-transfected with miR-195 mimics and MASCC1. Values and error bars are shown as the mean ± SD by one-way analysis of variance (ANOVA). (**D**) Representative images of wound healing showing the migration of CAL27 and SCC15 cells transfected with NC, miR-195 mimics, and MASCC1, or co-transfected with miR-195 mimics and MASCC1. Scale bar = 100 μm. (**E**) The percentage of wound healing in CAL27 and SCC15 cells transfected with NC, miR-195 mimics, and MASCC1, or co-transfected with miR-195 mimics and MASCC1. Values and error bars are shown as the mean ± SD by one-way ANOVA. * $p < 0.05$ and ** $p < 0.01$.

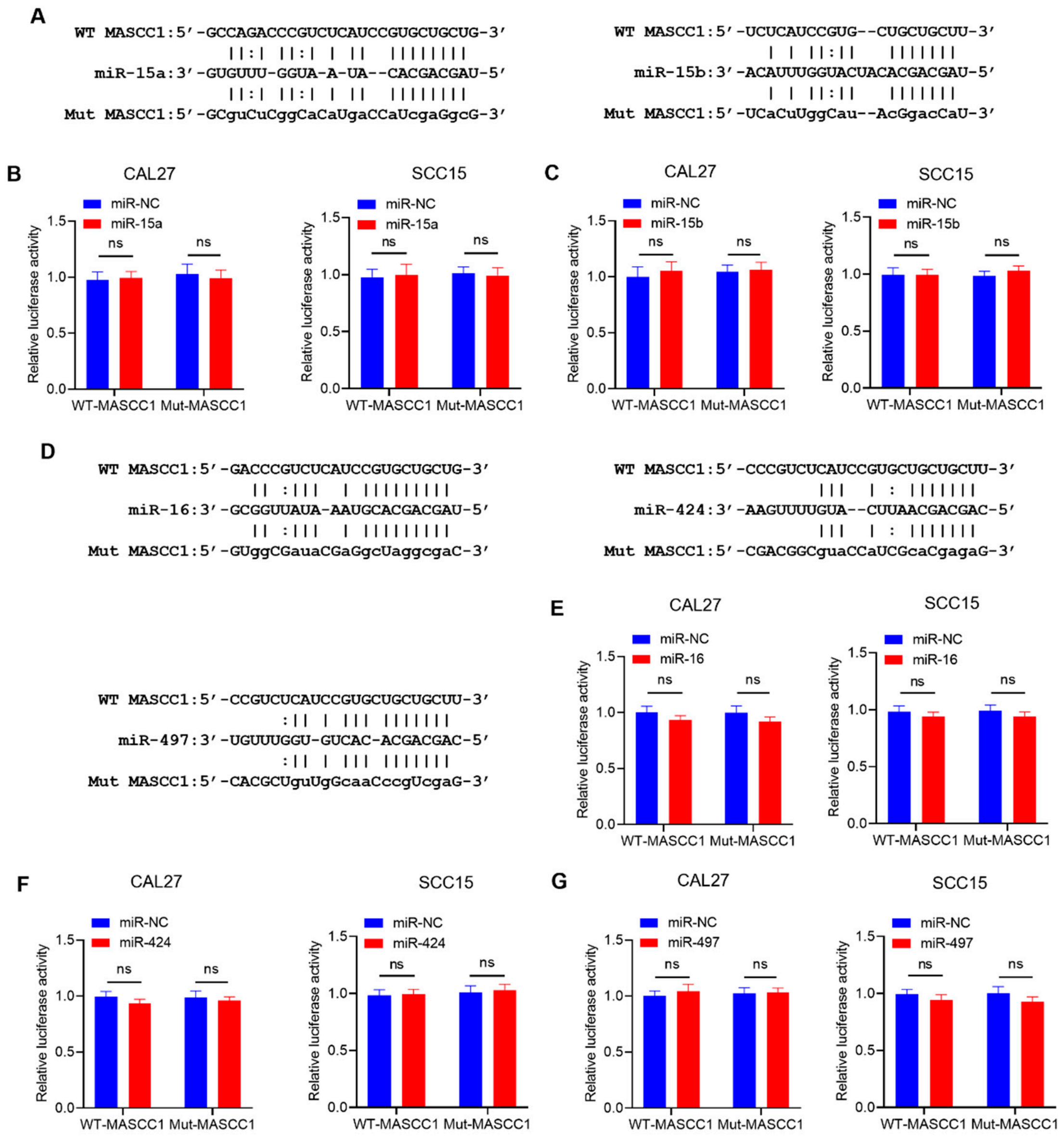

Figure S3. The miR-15/16 family binding sites on MASCC1. (**A**) The seed sequence of the miR-15 family (middle), including miR-15a and miR-15b, matches the binding sites of MASCC1 (WT; upper), along with mutations of the binding sites of MASCC1 (MUT; lower). (**B**,**C**) Histograms showing the effects of miR-15 family members, including miR-15a and miR-15b, on CAL27 and SCC15 cells with wild-type or mutated binding sites of MASCC1, reported as firefly luciferase activity. Values and error bars are shown as the mean ± SD with an unpaired Student's t-test. (**D**) The seed sequence of the miR-16 family (middle), including miR-16, miR-424, and miR-497, matches the binding sites of MASCC1 (upper), along with mutations of the binding sites of MASCC1 (lower). (**E**–**G**) Histograms showing the effects of miR-16 family members, including miR-16, miR-424, and miR-497, on CAL27 and SCC15 cells with wild-type or mutated binding sites of MASCC1, reported as firefly luciferase activity. Values and error bars are shown as the mean ± SD with an unpaired Student's t-test. ns, not significant.

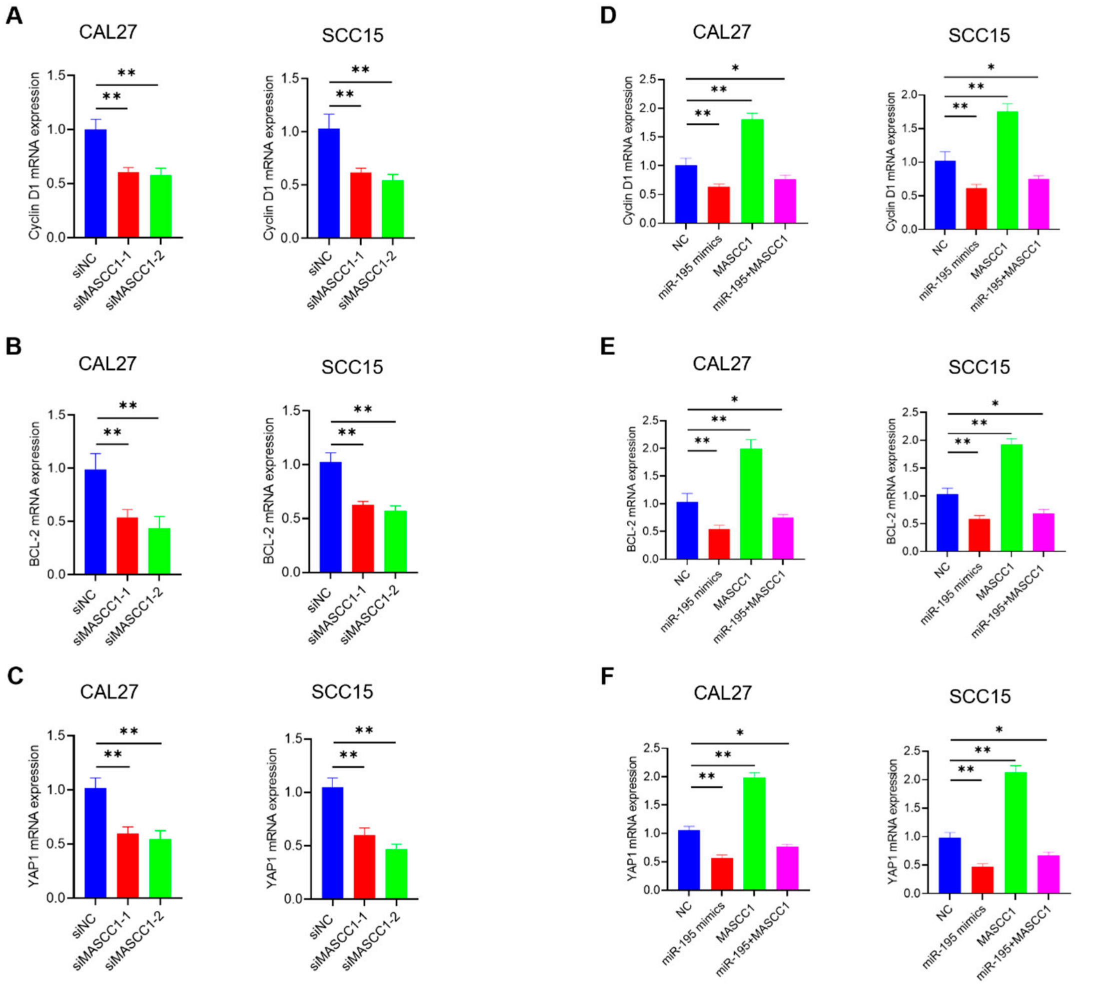

Figure S4. MASCC1 regulates the expression of Cyclin D1, BCL-2, and YAP1 mRNAs in HNSCC. (**A**–**C**) qRT-PCR analysis showing the relative mRNA expression of Cyclin D1, BCL-2, YAP1 in CAL27 and SCC15 cells with MASCC1 KD. Values and error bars are shown as the mean ± SD with an unpaired Student's t-test. (**D**–**F**) qRT-PCR analysis showing the relative mRNA expression of Cyclin D1, BCL-2, YAP1 in CAL27 and SCC15 cells transfected with the negative control (NC), miR-195 mimics, and MASCC1, or co-transfected with miR-195 mimics and MASCC1. Values and error bars are shown as the mean ± SD by one-way ANOVA. * $p < 0.05$ and ** $p < 0.01$.
