# Supplementary figures and images for: A Novel LncRNA MASCC1 Regulates the Progression and Metastasis of Head and Neck Squamous Cell Carcinoma by Sponging miR-195
Source: Cancers (Basel). 2023 Dec 11;15(24):5792. doi: 10.3390/cancers15245792 (PMC10741893; doi:10.3390/cancers15245792)

Figure 5C

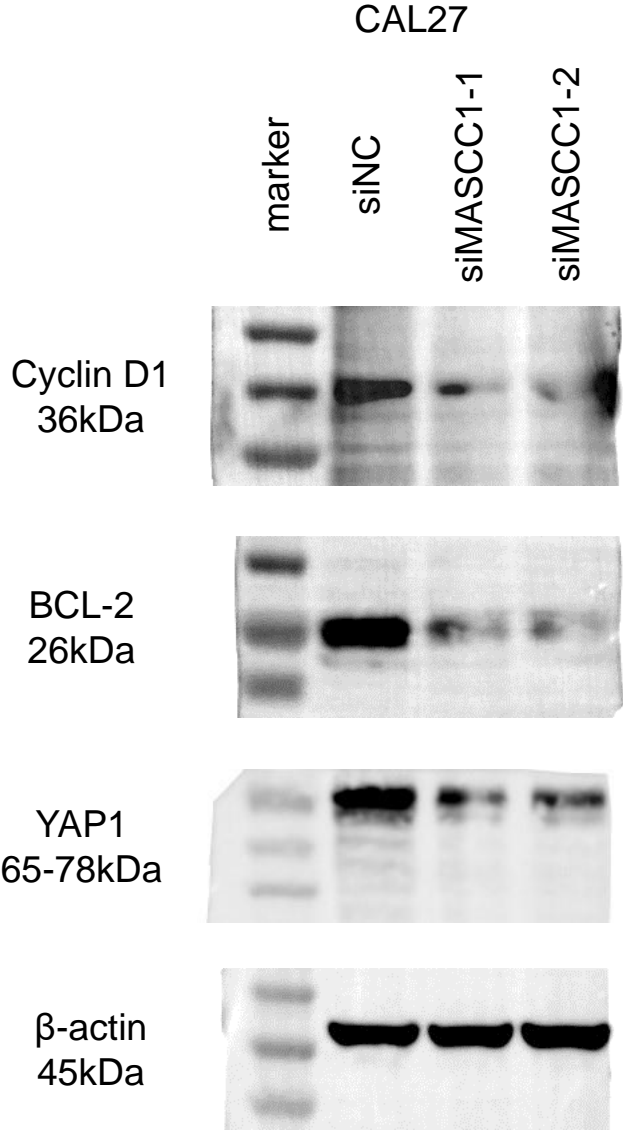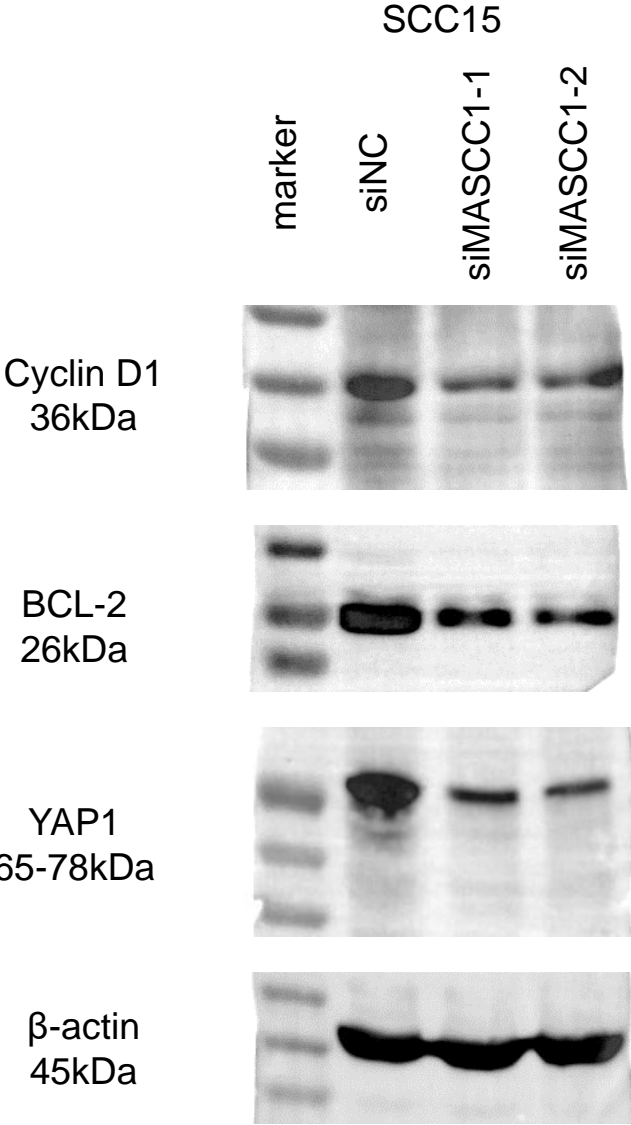

Figure 5G

CAL27

SCC15

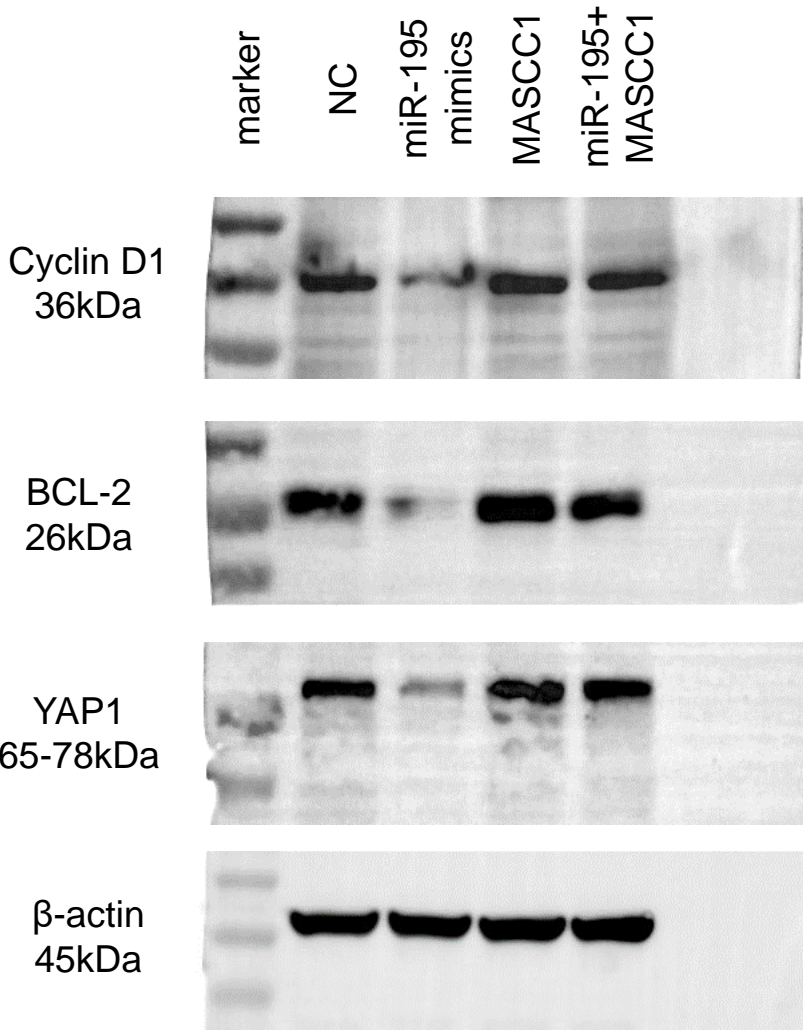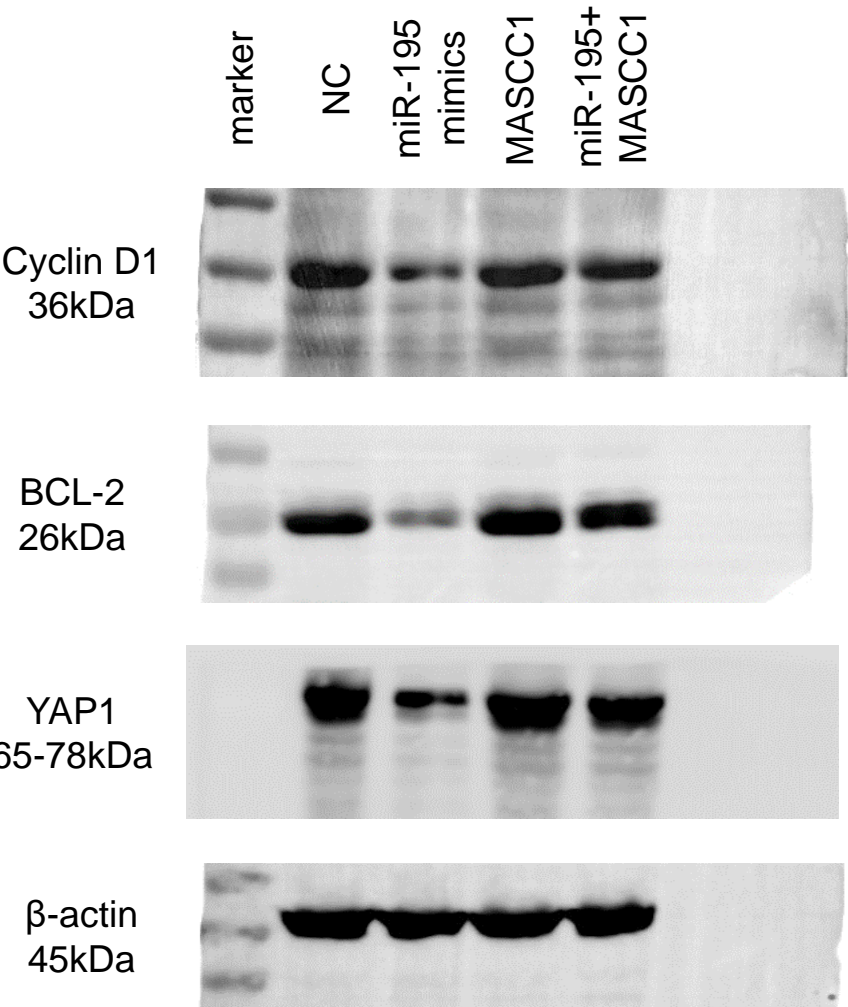

Supplement: Supplementary file 1 [file cancers-15-05792-s001.zip › Supplementary File S1. The uncropped blots of Figure 5C,G.pdf]
